# Plug-and-Display: decoration of Virus-Like Particles via isopeptide bonds for modular immunization

**DOI:** 10.1038/srep19234

**Published:** 2016-01-19

**Authors:** Karl D. Brune, Darren B. Leneghan, Iona J. Brian, Andrew S. Ishizuka, Martin F. Bachmann, Simon J. Draper, Sumi Biswas, Mark Howarth

**Affiliations:** 1 Department of Biochemistry, University of Oxford, South Parks Road, Oxford, OX1 3QU, UK; 2 Jenner Institute, University of Oxford, Oxford, OX3 7DQ, UK; 3 University Institute of Immunology, University of Bern, Sahli Haus 2, Inselspital, Bern, CH-3010, Switzerland

## Abstract

Virus-like particles (VLPs) are non-infectious self-assembling nanoparticles, useful in medicine and nanotechnology. Their repetitive molecularly-defined architecture is attractive for engineering multivalency, notably for vaccination. However, decorating VLPs with target-antigens by genetic fusion or chemical modification is time-consuming and often leads to capsid misassembly or antigen misfolding, hindering generation of protective immunity. Here we establish a platform for irreversibly decorating VLPs simply by mixing with protein antigen. SpyCatcher is a genetically-encoded protein designed to spontaneously form a covalent bond to its peptide-partner SpyTag. We expressed in *E. coli* VLPs from the bacteriophage AP205 genetically fused to SpyCatcher. We demonstrated quantitative covalent coupling to SpyCatcher-VLPs after mixing with SpyTag-linked to malaria antigens, including CIDR and Pfs25. In addition, we showed coupling to the VLPs for peptides relevant to cancer from epidermal growth factor receptor and telomerase. Injecting SpyCatcher-VLPs decorated with a malarial antigen efficiently induced antibody responses after only a single immunization. This simple, efficient and modular decoration of nanoparticles should accelerate vaccine development, as well as other applications of nanoparticle devices.

Virus-like particles (VLPs) are self-assembling multi-protein particles that mimic the structural organization and conformation of viruses. VLPs enable both surface display as well as payload encapsulation, providing versatility as biomedical research tools and vaccines^1^,^2^. Vaccines are one of the most effective of all medical interventions but for many diseases, such as malaria, HIV and cancer, vaccination has still had limited success^2^,^3^. Also, a rapid cycle of development, testing and production of new vaccines is required to deal with emerging pandemic viral threats^4^. Display on VLPs greatly enhances the response to otherwise poorly immunogenic epitopes^5^,^6^, because of a number of key characteristics. The dense protein arrangement of VLPs strongly triggers B-cell receptor clustering^6^,^7^. The 20–200 nm size of VLPs facilitates direct drainage to lymph nodes, increasing uptake by antigen-presenting cells and cross-presentation^6^,^8^. Also, the RNA or DNA packaged in VLPs triggers costimulatory signals through Toll-like receptors TLR3/7/8/9^7^,^8^. VLPs can be conveniently produced at high quantities and commercial scale through heterologous recombinant gene expression in a range of expression systems, including *Escherichia coli*, tobacco and yeast species^9^. VLPs are in use or under development to vaccinate against bacterial, viral and protozoal pathogens^3^,^10^,^11^, as well as against chronic diseases (autoimmunity, allergy, neurodegenerative diseases and cancer)^12^,^13^,^14^.

Display of antigens on VLPs is usually achieved by either chemical conjugation or genetic fusion to the viral coat protein^1^,^15^. Genetic fusion has been successful in numerous examples but remains a time-consuming trial-and-error approach^16^. Genetic fusion of certain antigens leads to misfolding of the antigen or the capsid; this misfolding either impairs VLP assembly or stability, or leads to the display of antigen in a non-native conformation, so that the antibodies raised are ineffective^15^,^17^,^18^. Another major challenge with genetic fusion is that the host optimal for VLP expression and the host optimal for antigen expression (bacterium, yeast, mammalian cell-line, or plant) may have different post-translational modifications (particularly disulfide bond formation and glycosylation)^15^,^19^.

Therefore, separately expressing the VLP and target antigen is often preferable or necessary; subsequently the VLP and target antigen must be linked together. Conjugation through the abundant amino groups (Lys, N-terminus) or carboxyl groups (Asp, Glu, C-terminus) gives a heterogeneous mixture, so conjugation through an exposed sulfhydryl group (Cys) on the antigen is the most common route^19^,^20^. Chemical conjugation may give limited control over the number of VLP modifications, leading to heterogeneous and incomplete VLP coverage with antigen^18^,^21^. Also, many surface-exposed antigens of pathogenic or human origin contain disulfide bonds, which play an important role in protein folding and stabilization, so a new cysteine can be disruptive^22^,^23^.

Alternative routes to VLP functionalization include non-covalent VLP:antigen linkage, such as attaching Ni-NTA to VLPs for linkage of His-tagged antigens^24^ but the stability during storage and in the body may be an issue. Glycosylphosphatidylinositol (GPI)-anchored proteins can be expressed in one cell-type and then transferred into VLPs, although this is restricted to VLPs containing a lipid bilayer and the purification of the GPI-linked proteins is non-trivial^14^.

Unnatural amino acids may be incorporated into the VLP and antigen for click chemistry conjugation^25^,^26^,^27^, enabling site-specific modification and stable linkage, but the increased complexity of protein expression, side-reactions of certain azide/alkyne/tetrazine groups^28^,^29^,^30^ and the increased frequency of misreading of unnatural amino acid codons^31^ mean that there is still a need for simple and scalable approaches for covalent VLP:antigen linkage.

To address this challenge, we made use of genetically-encoded spontaneous amide bond formation^32^. SpyTag is a peptide we previously engineered to form a spontaneous and irreversible isopeptide bond to its protein partner SpyCatcher simply upon mixing (Fig. 1)^33^. Both SpyTag and SpyCatcher can be positioned at various locations in protein chains, are reactive under a wide range of conditions (pH, buffer, temperature), and do not possess cysteines^33^,^34^,^35^. Here we establish the use of SpyTag-SpyCatcher reactivity for modular VLP functionalization, characterizing SpyCatcher-linked VLPs, demonstrating conjugation to a range of different antigens, and showing effective immunization in mice.

## Results

### SpyCatcher-VLP expression and analysis

**Figure 1:**
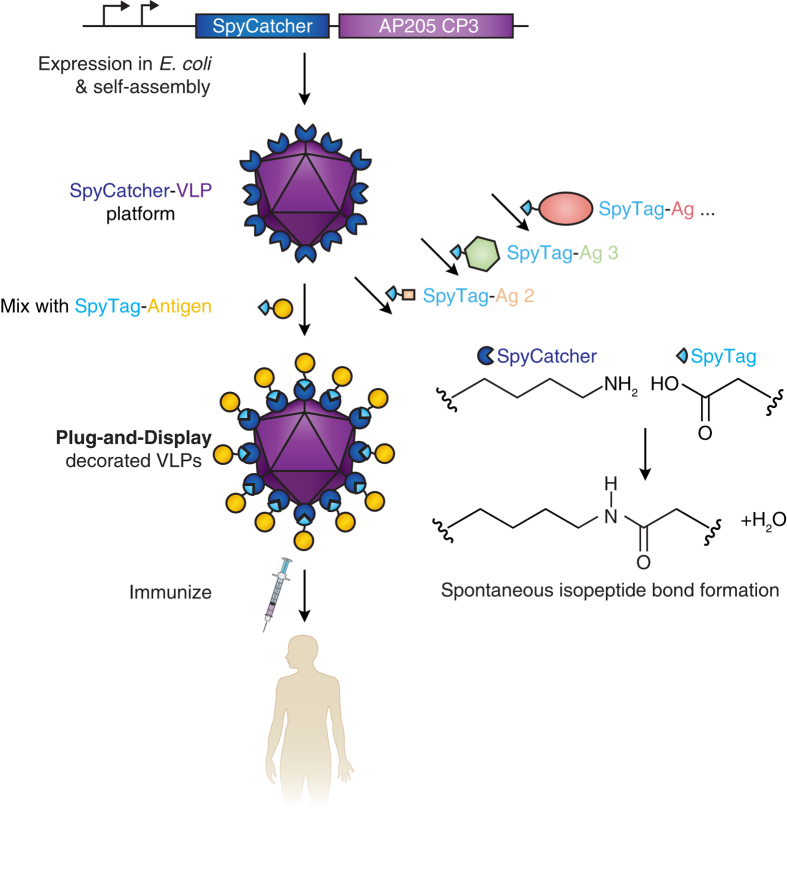
Overview of Plug-and-Display VLP assembly. SpyCatcher is genetically fused to the AP205 phage coat protein (AP205 CP3) and expressed in *E. coli*. Self-assembly of monomers generates SpyCatcher-VLPs. Upon mixing, SpyTag-antigen forms a spontaneous isopeptide bond with SpyCatcher-VLPs, yielding decorated particles for immunization.

**Figure 2:**
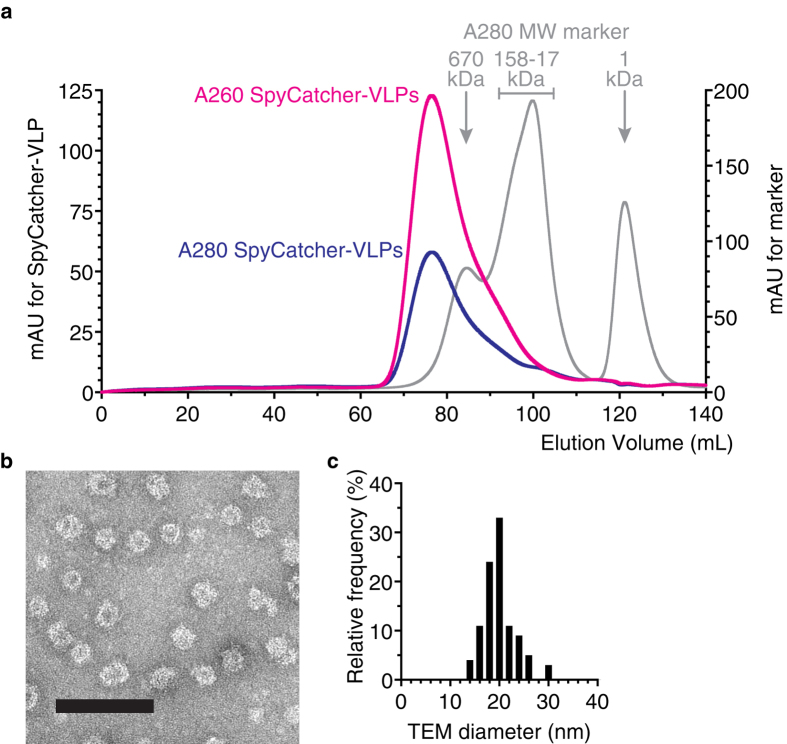
Characterization of SpyCatcher-VLP assembly. (**a**) Size-exclusion chromatography showed SpyCatcher-CP3 assembly into VLPs, analyzed by absorbance at 260 and 280 nm and compared to Mw markers. (**b**) Negatively-stained TEM image of SpyCatcher-VLPs. Scale bar 100 nm. (**c**) Size distribution of SpyCatcher-VLPs from TEM (n = 100).

We genetically fused SpyCatcher to the N-terminus of the viral coat protein (CP3) of the RNA bacteriophage AP205 (Fig. 1)^36^,^37^. SpyCatcher-CP3 was expressed in *E. coli* and VLPs were purified to homogeneity from cell-lysate by Ni-NTA affinity chromatography and then by dialysis using a 300 kDa molecular weight cut-off (MWCO) membrane. Size-exclusion chromatography showed the material eluting in the MDa range expected for VLPs, with concurrent elution of protein (280 nm absorbance) and nucleic acid species (260 nm absorbance), typical for AP205 VLPs (Fig. 2a)^36^,^38^,^39^. Negative-staining transmission electron microscopy (TEM) revealed the presence of particles consistent with VLPs (Fig. 2b). Quantifying the particle size from TEM gave a diameter of 20 ± 3.2 nm (mean ± 1 s.d., n = 100) (Fig. 2c).

### SpyCatcher-VLP covalent conjugation

**Figure 3:**
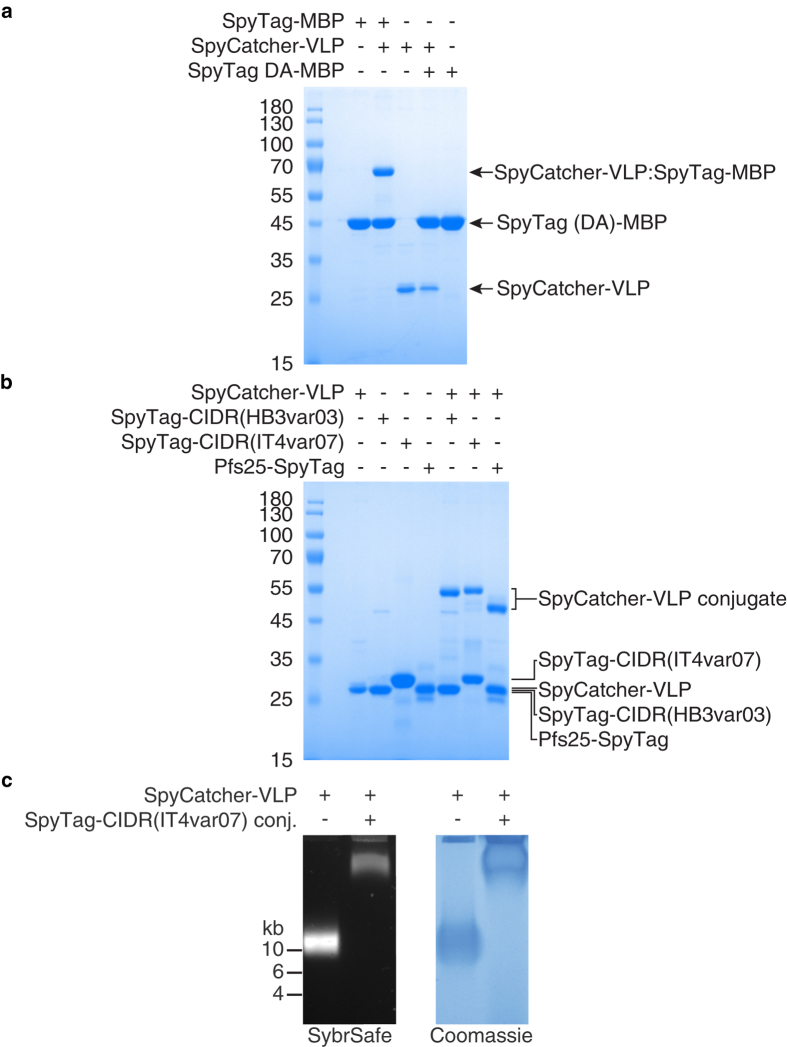
Decoration of VLPs by spontaneous isopeptide bond formation. (**a**) Complete reaction of SpyCatcher-VLPs with SpyTag. SpyCatcher-VLPs were incubated with SpyTag-MBP or the negative control SpyTag DA-MBP, before boiling in SDS-loading buffer and analysis by SDS-PAGE with Coomassie staining. (**b**) SpyCatcher-VLP reacted with malarial protein antigens. As in (a), except with SpyTag-CIDR(HB3var03), SpyTag-CIDR(IT4var07) or Pfs25-SpyTag. (**c**) Native agarose gel electrophoresis of VLPs. The gel was stained with SybrSafe (nucleic acid) and then Coomassie dye (protein), showing unconjugated SpyCatcher-VLPs and SpyCatcher-VLPs conjugated with SpyTag-CIDR(IT4var07).

For initial validation of SpyCatcher-VLP reactivity, we mixed the VLPs with the model SpyTag-linked protein, *E. coli* maltose binding-protein (MBP)^33^. SpyCatcher-CP3 gave a clean band on SDS-PAGE and after mixing with excess SpyTag-MBP, all the SpyCatcher-CP3 moved to a higher molecular weight, consistent with quantitative covalent bond formation (Fig. 3a). The negative control SpyTag DA-MBP, with the reactive aspartic acid of SpyTag changed to alanine^33^, did not change SpyCatcher-CP3 mobility (Fig. 3a).

We then tested SpyCatcher-VLP decoration with immunologically-relevant antigens from *Plasmodium falciparum*. *P. falciparum* erythrocyte membrane protein 1 (PfEMP1) contains a Complex lysine and cysteine-rich Inter-Domain Region (CIDR). CIDR interaction with endothelial protein C receptor (EPCR) is associated with severe childhood malaria, through the trapping of parasite-infected red blood cells in the brain microvasculature^40^. HB3var03 and IT4var07 represent two distinct versions of the CIDR domain from different natural isolates, with similar structures but substantial sequence divergence^40^.

SpyTag was genetically fused to the N-terminus of each CIDR antigen, expressed and purified from *E. coli*, and incubated with SpyCatcher-VLPs. Complete reaction was seen to SpyTag-CIDR(IT4var07) (Fig. 3b). Pfs25 is a *P. falciparum* sexual-stage antigen displayed on the surface of the parasite at the zygote and ookinete stages. Pfs25 is a leading malaria transmission-blocking candidate antigen: antibodies against Pfs25 completely prevent development of the parasite in the mosquito^41^,^42^. SpyTag was genetically fused to the C-terminus of Pfs25, before expression in mammalian cells. SpyCatcher-VLPs also reacted covalently with Pfs25-SpyTag (Fig. 3b). These results confirmed that SpyCatcher-VLPs were efficient for conjugation to protein antigens linked to SpyTag at either terminus and expressed in bacteria or eukaryotic cells.

We also tested conjugation of SpyCatcher-VLPs to solid-phase synthesized peptide antigens, in this case relevant to cancer immunity. The Telo peptide is based on a human telomerase reverse transcriptase mutant epitope, tested for immunization against a range of cancers^43^. The EGFRvIII peptide is derived from the cancer-specific epidermal growth factor receptor (EGFR) EGFRvIII mutant sequence, applied for immunization against human glioblastoma^44^. High yielding conjugation to SpyCatcher-VLPs was observed after incubating with SpyTag-Telo and EGFRvIII-SpyTag (Supplementary Fig. S1).

Native agarose gel electrophoresis was used to test that covalent reaction occurred while the coat protein was assembled in VLPs. Imaging of the gel showed co-migration of a distinct protein band and nucleic acid band, indicating encapsulation of nucleic acid by the hybrid SpyCatcher-VLPs (Fig. 3c). AP205 VLPs have been previously shown to encapsulate various RNAs from their expression host^39^,^45^. This SpyCatcher-VLP band was uniformly decreased in mobility by conjugation with SpyTag-CIDR(IT4var07) (Fig. 3c). As in subsequent experiments, excess antigen was simply removed from conjugated VLPs by dialysis using a high MWCO membrane.

### Immunization using SpyCatcher-VLPs against CIDR

**Figure 4:**
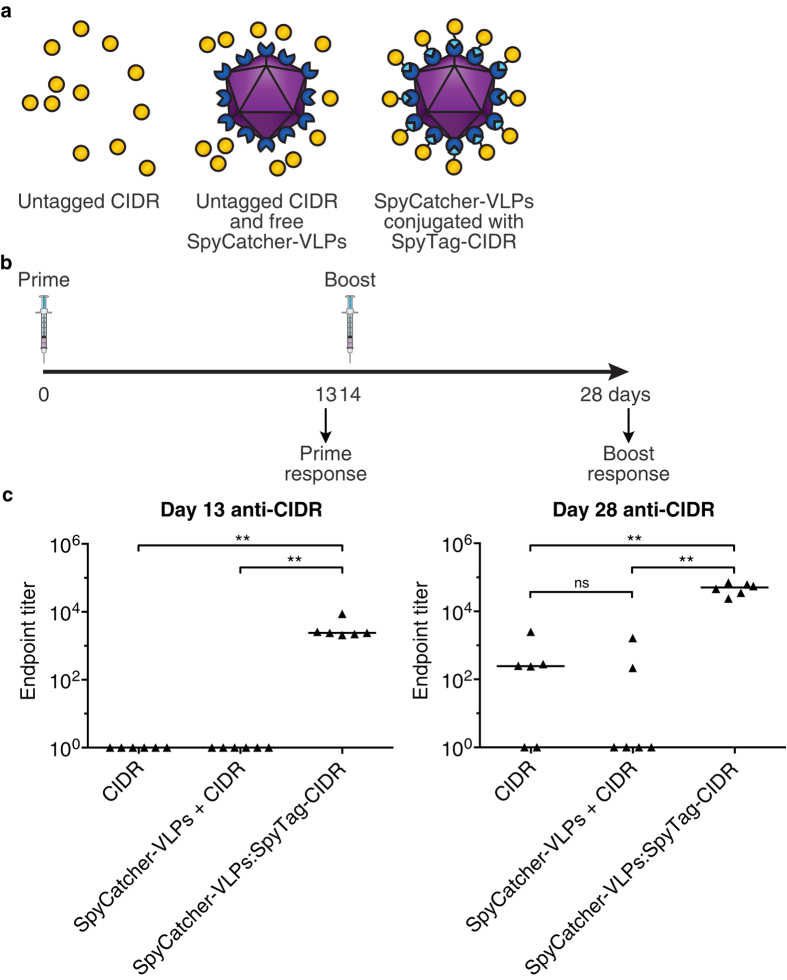
Plug-and-Display immunization against CIDR. (**a**) Schematic of immunization route with SpyCatcher-VLPs covalently decorated with SpyTag-CIDR(IT4var07) or the negative controls: SpyCatcher-VLPs + untagged CIDR (no covalent association) or CIDR alone. (**b**) Time-course of immunization and sampling. (**c**) Antibody response to CIDR after Plug-and-Display immunization. 6 mice per condition were immunized on days 0 and 14 with CIDR, SpyCatcher-VLPs + untagged CIDR, or SpyCatcher-VLPs conjugated to SpyTag-CIDR. After 13 days (left) or 28 days (right), the total IgG anti-CIDR titer was determined by ELISA. Triangles represent the value for each mouse, while the horizontal bar represents the median. ns not significant, **p < 0.01, determined by Mann-Whitney test (n = 6).

Having established the integrity and reactivity of the particles, we explored the efficiency of SpyCatcher-VLPs for generating an immune response. We focused on the immune response to CIDR(IT4var07). Mice were immunized intramuscularly with 20 μg CIDR(IT4var07) equivalent, either as SpyCatcher-VLPs:SpyTag-CIDR conjugate, a negative control of SpyCatcher-VLPs + untagged CIDR, or a further negative control of CIDR alone (Fig. 4a). Reaction to form the SpyCatcher-VLPs:SpyTag-CIDR conjugate was validated by SDS-PAGE (Supplementary Fig. S2a). All immunizations were performed in the absence of adjuvant. Mice were boosted at day 14 with the same dosing regime. The antibody titer against CIDR was measured 2 weeks after each immunization (on days 13 and 28) via enzyme-linked immunosorbent assay (ELISA) (Fig. 4b). At each time-point tested, SpyCatcher-VLPs:SpyTag-CIDR induced a higher anti-CIDR response than either SpyCatcher-VLPs + untagged CIDR or the CIDR-only group (Fig. 4c). At day 13, only the SpyCatcher-VLPs:SpyTag-CIDR group showed anti-CIDR antibody generation, significantly better than with SpyCatcher-VLPs + untagged CIDR or the CIDR-only (in each case p = 0.002, n = 6, Mann-Whitney test) (Fig. 4c). After boosting, an anti-CIDR antibody response was seen in 6 of 6 mice in the SpyCatcher-VLPs:SpyTag-CIDR group, but only in 2 of 6 mice with SpyCatcher-VLPs + untagged CIDR and 4 of 6 mice with CIDR-only, indicating that, as expected, on its own CIDR is a poor immunogen (Fig. 4c). The anti-CIDR response at day 28 for the SpyCatcher-VLPs:SpyTag-CIDR group was significantly higher than the responses of the SpyCatcher-VLPs + untagged CIDR group or the CIDR-only group (in each case p = 0.002, n = 6, Mann-Whitney test) (Fig. 4c). The presence of unconjugated SpyCatcher-VLPs did not affect the immune response to CIDR (day 28 p = 0.3, not significant, n = 6, Mann-Whitney test).

To measure the immune response against the SpyCatcher-VLP platform, rather than the antigen target, we coated ELISA plates with SpyCatcher-VLPs and determined the antibody titer. As expected, mice in the SpyCatcher-VLPs:SpyTag-CIDR and SpyCatcher-VLPs + untagged CIDR groups generated an immune response to SpyCatcher-VLPs (Supplementary Fig. S3). However, this response to SpyCatcher-VLPs at day 13 was significantly lower in the SpyCatcher-VLPs:SpyTag-CIDR group than the SpyCatcher-VLPs + untagged CIDR group (p = 0.002, n = 6, Mann-Whitney test). Boosting with either conjugated or unconjugated particles increased the antibody response to SpyCatcher-VLPs by approximately 1 log, but again the response in the SpyCatcher-VLPs:SpyTag-CIDR group was significantly lower than the SpyCatcher-VLPs + untagged CIDR group (p = 0.002, n = 6, Mann-Whitney test) (Supplementary Fig. S3), consistent with masking of the SpyCatcher-VLP surface by the covalently conjugated CIDR.

### Immunization using SpyCatcher-VLPs against Pfs25

**Figure 5:**
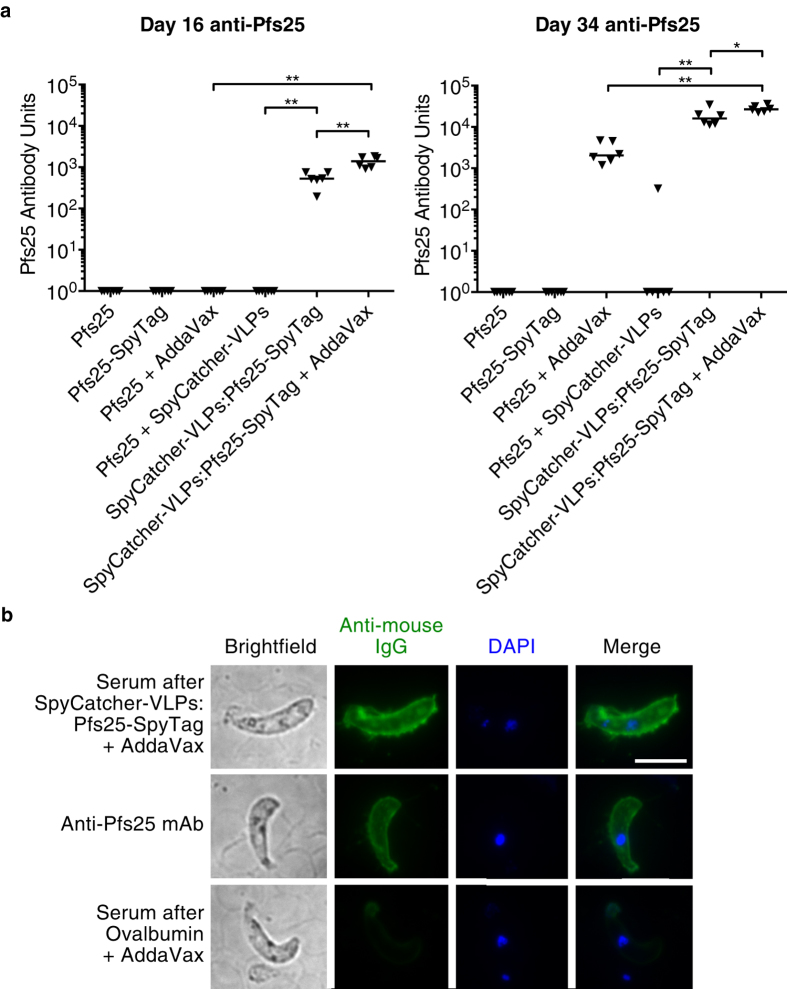
Plug-and-Display immunization against Pfs25. (**a**) Antibodies were raised to Pfs25 after SpyCatcher-VLP immunization. 6 mice per condition were immunized on day 0 and 17 with Pfs25, Pfs25-SpyTag, Pfs25-SpyTag conjugated to SpyCatcher-VLPs, or SpyCatcher-VLPs + untagged Pfs25. Where indicated, the adjuvant AddaVax was included. After prime (16 days, left) or boost (34 days, right), total anti-Pfs25 IgG was determined by ELISA (expressed as Pfs25 Antibody Units, by comparison to reference serum). Triangles represent the value for each mouse, while the horizontal bar represents the median. *p < 0.05, **p < 0.01, determined by Mann-Whitney test (n = 6). (**b**) Antibodies from SpyCatcher-VLPs:Pfs25-SpyTag immunization bound the ookinete surface. Ookinetes expressing Pfs25 were stained with day 34 serum from a mouse immunized with SpyCatcher-VLPs:Pfs25-SpyTag and AddaVax (top row). As a positive control, cells were stained with a monoclonal antibody against Pfs25 (middle row). As a negative control, cells were stained with serum from a mouse immunized with ovalbumin and AddaVax (bottom row). Cells were visualized in the fluorescence microscope, shown with brightfield imaging (grayscale, left), antibody staining (green, center left), DAPI DNA stain (blue, center right), and an overlay of DAPI and antibody staining (right). Scale bar = 5 μm.

To support further the applicability of the SpyCatcher-VLP platform, we performed immunization against Pfs25, a promising transmission-blocking antigen^41^. We immunized mice intramuscularly with doses matched to 2.5 μg of each version of Pfs25. Immunizing with Pfs25 and Pfs25-SpyTag was to test whether SpyTag itself somehow enhanced the immunogenicity of a linked antigen. These two proteins were then also injected with SpyCatcher-VLPs. Reaction of SpyCatcher-VLP with Pfs25-SpyTag for immunization was validated by SDS-PAGE (Supplementary Fig. S2b). We also tested how adjuvant affected the antibody response. AddaVax, a squalene-based oil-in-water nanoemulsion, is a potent adjuvant, reported to promote a balanced Th1/Th2 immune response and based on the MF59 adjuvant licensed for use in an influenza vaccine^46^. Mice were boosted at day 17 with the same dosing regime. The antibody titer against Pfs25 was measured on days 16 and 34 via ELISA (Fig. 5a).

Pfs25 and Pfs25-SpyTag proteins alone did not give a response, even after boosting. Pfs25 with AddaVax gave a response only after boosting. SpyCatcher-VLPs conjugated to Pfs25-SpyTag gave a strong response even before boosting, whereas the negative control of SpyCatcher-VLPs + untagged Pfs25 gave significantly less response at day 16 or 34 (in each case p = 0.002, n = 6, Mann-Whitney test). Including adjuvant gave a small increase to the SpyCatcher-VLPs:Pfs25-SpyTag response after the prime (p = 0.002, n = 6, Mann-Whitney test) and after the boost (p = 0.04, n = 6, Mann-Whitney test) (Fig. 5a). Even with adjuvant, the response to Pfs25 protein did not match the response to SpyCatcher-VLPs:Pfs25-SpyTag (for day 16 or 34, p = 0.002, n = 6, Mann-Whitney test).

To test further whether the antibodies raised from the SpyCatcher-VLP immunization could recognize native antigen, we stained a blood smear containing ookinetes expressing Pfs25 on their surface and detected the antibody staining by fluorescence microscopy (Fig. 5b). Serum from mice boosted with SpyCatcher-VLPs:Pfs25-SpyTag and AddaVax gave strong antibody labeling on the surface of the ookinete, similar to the pattern with the validated anti-Pfs25 monoclonal antibody 4B7. As a negative control, serum from a mouse immunized with the control antigen ovalbumin and AddaVax gave minimal surface staining (Fig. 5b).

## Discussion

We have established a platform for simple, rapid and stable linking of antigens to virus-like particles for immunization. We demonstrated VLP decoration with a range of malarial protein antigens and cancer-related peptides. This is consistent with the tolerance for SpyTag/SpyCatcher fusion shown by ourselves and other laboratories for applications with dendritic cell-targeting antibodies^47^, membrane protein imaging^48^, biofilms^49^,^50^ and hydrogels^51^. SpyCatcher-VLPs could be simply expressed from *E. coli*. For isolated Pfs25 protein, we required adjuvant and boosting to obtain a robust antibody response. SpyCatcher-VLPs decorated with the CIDR or Pfs25 antigens generated a robust antibody response after only a single immunization without requiring adjuvant.

In future work it will be interesting to explore the use of SpyCatcher-SpyTag ligation for decoration of other vaccine-relevant platforms, such as norovirus and Hepatitis B virus^3^,^24^,^52^,^53^. The recently approved malaria vaccine still has only modest efficacy^54^ so further malaria vaccine engineering is clearly needed. It will be useful to test how the immune response from the SpyCatcher-VLP platform relates to disease protection in animal challenge models, including with promising malarial transmission-blocking antigens like Pfs25^41^,^55^ or blood-stage antigens like RH5^52^,^56^.

SpyCatcher is derived from *Streptococcus pyogenes* and the coat protein is from a bacteriophage, so antibodies were raised as expected to the SpyCatcher-VLP platform, although coupling the antigen reduced this immunogenicity^47^. Most VLPs are immunogenic, so this is not a new issue; indeed VLP carriers can be used for repeated booster immunizations and existing antibody responses against the vaccine may even benefit vaccine immunogenicity at the time of boosting. The RTS,S malaria vaccine, based on the Hepatitis B surface antigen (HBsAg) backbone, has been given successfully three times with a booster immunization at 20 months whilst also inducing anti-HBsAg antibody responses^57^. This is in contrast to live viral vaccine vectors which require *in situ* antigen expression following infection of immunized cells; in this case anti-vector immunity impedes subsequent vector administration, meaning that different immunization platforms/vectors are required for prime versus boost^3^,^52^. Even with antibodies to SpyCatcher-VLPs present at day 13, we saw a second injection with conjugated SpyCatcher-VLPs still boosted the response to the target CIDR antigen. The Tag/Catcher principle of covalent peptide reaction has been established for a number of different pairs and so it may be possible to use these other pairs in future rounds of immunization^58^,^59^. Apart from vaccination, it will also be valuable to explore the use of Plug-and-Display nanoparticle decoration in other applications, such as drug delivery, enzyme scaffolds, biosensors, and cancer immunotherapy^1^,^60^,^61^.

## Methods

### Cloning

Expression constructs were cloned using standard PCR methods, Gibson isothermal assembly^62^ or In-Fusion HD Cloning Plus kit (ClonTech) and inserts were verified by Sanger sequencing. pGEM-SpyCatcher-AP205cp3 was used to generate SpyCatcher-VLPs and has the organization: N-terminal His_6_, N-terminally truncated ∆N1SpyCatcher^34^, (GSG)_3_ spacer, AP205 coat protein 3^36^ (codon-optimized for expression in *E. coli* by GeneArt) (GenBank accession number KU302810). pET28a-SpyTag-MBP and pET28a-SpyTag DA-MBP have been described^33^. pET15b-CIDR(IT4var07) has the organization: N-terminal His_6_, tobacco etch virus (TEV) cleavage site, GSG linker, *P. falciparum* CIDR(IT4var07)^40^. pET15b-SpyTag-CIDR(HB3var03) has SpyTag at the N-terminal side of the *P. falciparum* CIDR(HB3var03)^40^. pET15b-SpyTag-CIDR(IT4var07) has the organization: N-terminal His_6_, TEV cleavage site, GGS linker, SpyTag, GGS linker, *P. falciparum* CIDR(IT4var07, starting EPAPDVKT…)^40^. pENTR4-Pfs25-SpyTag^63^ for expression in mammalian cells has a signal peptide from tissue plasminogen activator before *P. falciparum* Pfs25 with C-terminal SpyTag, followed by C-tag for purification^64^ (GenBank accession number KU302811).

pFH255, a kind gift of Ario de Marco (University of Nova Gorica), has Erv1p (sulfhydryl oxidase) and DsbC (disulfide bond isomerase) expressed under arabinose control for enhanced cytosolic formation of disulfide bonds^35^,^65^. His_6_-TEV protease expression vector was a kind gift of the Stephen Bottomley laboratory, Monash University, and has the organization: MBP-TEV_site-His_6_-TEV_protease (L56V, S135G, S219V)-R_5_^66^.

### Expression of SpyCatcher-VLPs

C41 *E. coli*^67^, a kind gift of Anthony Watts (University of Oxford), was transformed with pGEM-SpyCatcher-AP205cp3 and pFH255 and plated on LB-Lennox (5 g/L NaCl) agar plates with 100 μg/mL ampicillin and 34 μg/mL chloramphenicol. The plates were incubated at 37 °C for 16–20 h. 4 entire colonies were picked into 400 mL LB-Lennox medium with 20 μg/mL ampicillin and 34 μg/mL chloramphenicol in a 2 L strongly-baffled glass flask and incubated without shaking for 10–14 h at 37 °C. The culture was then diluted 1:1 with fresh media (final concentration 20 μg/mL ampicillin, 17 μg/mL chloramphenicol assuming that all previous ampicillin was degraded overnight) and incubated with shaking at 120 rpm (Infors Multitron, 2.5 cm throw) at 37 °C for 1 h, before induction at A600 0.9 with 1 mM IPTG for 6–8 h at 37 °C, shaking at 180 rpm.

### Purification of VLPs

One 1,000 mL culture-derived pellet (typically 12 in total) was washed in 50 mL ice-cooled PBS and spun for 10 min at 4,000 *g* at 4 °C. The pellet was resuspended at RT in 10 mL lysis buffer: 20 mM Tris•HCl, 150 mM NaCl, 0.1% (v/v) Triton X-100, 0.1% (v/v) Tween 20, pH 7.8. The resuspended mixture was incubated at RT for 15 min, placed in an ice-salt water bath for 10 min and then sonicated 4 times for 30 s, with a minimum of 1 min between each pulse. The lysates were spun twice at 15,000 *g* for 20 min at 4 °C. The supernatant was filtered using a 1.2 μm glass filter device (Sartorius) and then through a 0.45 μm surfactant-free cellulose acetate filter device (Nalgene). 250 U benzonase (Sigma) was added and the mixture incubated for 5 min at RT. Ni-NTA elution buffer (50 mM Tris•HCl, 300 mM NaCl, 1 M imidazole, pH 7.8) was then added to a final concentration of 75 mM imidazole. 1 mL of packed Ni-NTA agarose (Qiagen) was added to the cleared lysate. The tubes were incubated on a rotary shaker for 10 min at 4 °C and then centrifuged for 2 min at 4,700 *g*, 4 °C. The resin was washed three times in 100 mM imidazole washing buffer. The supernatant was then aspirated and the remaining resin was once more added to the cleared lysate imidazole-conditioned solution, the process repeated and finally resuspended in 15 mL 100 mM NTA-buffer. 5 mL each were transferred to a 15 mL Falcon tube (3 in total) and each tube was topped up to 15 mL with 100 mM washing buffer containing 0.1% (v/v) Triton X-114 (TX114), to remove endotoxin^68^. Two more washes with full volume TX114, 100 mM imidazole were applied, prior to three washes without TX114. The sample was resuspended and applied to a polyprep column. For elution, the polyprep column was capped, 2.5 mL of elution buffer [2 M imidazole, 50 mM glycine, 25 mM sodium citrate, 0.1% (v/v) Tween 20, pH 8.5] added and the resin incubated on a mini shaker (900 rpm) for 10 min at RT prior to elution. The eluate was once more cleared over a mini polyprep column (Bio-Rad) to remove any agarose resin. The flow-through in 1.5 mL centrifuge tubes was spun for 10 min at 4 °C at 17,000 *g*, pipetted into a 300 kDa MWCO cellulose ester dialysis tubing (SpectrumLabs) and dialyzed overnight at 4 °C against 1,000-fold excess of 50 mM glycine, 25 mM sodium citrate, 0.1% (v/v) Tween 20, pH 8.0 for buffer exchange and to deplete species such as VLP monomer. Dialysis was repeated in fresh buffer for an additional 3 h. After dialysis, the sample was spun once more at 17,000 *g* for 10 min at 4 °C, to remove any aggregate.

### Expression of CIDR proteins

ClearColi BL21(DE3) (Lucigen), which have mutated lipopolysaccharide for low endotoxin protein production^69^, was transformed with the plasmid pSC101 argU ileY leuW StrepR/SpecR from BL21-CodonPlus(DE3)-RIPL (Agilent) and pFH255. The bacteria were plated onto 34 μg/mL chloramphenicol and 60 μg/mL streptomycin, yielding ClearColi BL21(DE3)-RIL-dsbC-erv1p. Single colonies were obtained after ~36 h and CaCl_2_-competent cells were prepared. pET15b-CIDR(IT4var07), pET15b-SpyTag-CIDR(HB3var03) or pET15b-SpyTag-CIDR(IT4var07) was transformed into ClearColi BL21(DE3)-RIL-dsbC-erv1p and selected on LB-Miller (10 g/L NaCl), 34 μg/mL chloramphenicol, 100 μg/mL ampicillin. Singe colonies (visible after 36–48 h) were inoculated into LB-Miller 100 μg/mL ampicillin, 20 μg/mL chloramphenicol, 30 μg/mL streptomycin for 15 h at 37 °C with shaking at 180 rpm with 2.5 cm throw. The cultures were diluted 1:100 into 750 mL LB in 2 L strongly baffled glass flasks (Scientific Laboratory Supplies), grown to A600 0.6, induced with 2.5 g arabinose, except for pET15b-SpyTag-CIDR(HB3var03), grown as before until A600 0.7–1.0 and then induced with 1 mM IPTG for 12–14 h at 22 °C, shaking at 250 rpm prior to harvest.

### Purification of CIDRs

Each cell pellet was resuspended in 12 mL lysis buffer: 20 mM Tris•HCl, 300 mM NaCl, 15 mM imidazole, 0.5% (v/v) Triton X-100, 10% (v/v) glycerol, 1 mg/mL lysozyme (Sigma), and 2 U/mL benzonase pH 8.0. The sample was sonicated on ice using 4 × 30 s pulses, with 1 min rest between each pulse. The insoluble fraction was spun down at 27,000 *g* at 4 °C. For pET15b-CIDR(IT4var07) and pET15b-SpyTag-CIDR(IT4var07), the soluble protein in the supernatant was purified using standard Ni-NTA as above, with washing at 30 mM imidazole and elution at 200 mM imidazole. pET15b-SpyTag-CIDR(HB3var03) was purified from inclusion bodies, as described^40^. Briefly, inclusion bodies were resuspended in guanidinium hydrochloride, bound to Ni-NTA and refolded on resin with decreasing guanidinium concentration. Protein was then eluted with Ni-NTA elution buffer (200 mM imidazole, 20 mM Tris•HCl, 300 mM NaCl, pH 8.0). His_6_-TEV protease^66^ purified in redox buffer (20 mM Tris•HCl, 300 mM NaCl, 3 mM reduced glutathione, 0.3 mM oxidised glutathione, pH 8.0) from ClearColi BL21(DE3)-RIPL His_6_-TEV was added at 1:100 A280 equivalents to protein derived from pET15b-CIDR(IT4var07) and pET15b-SpyTag-CIDR(IT4var07). TEV protease digestion to remove the N-terminal His_6_ tag for ELISA was performed in the presence of redox buffer for 4 h at 30 °C. The processed eluate was then dialyzed twice at 4 °C against 1,000-fold excess 20 mM Tris•HCl, 300 mM NaCl, pH 7.8 with a 3.5 kDa cut-off membrane (Fisher Scientific) and applied to 0.5 mL of binding buffer (20 mM Tris•HCl, 150 mM NaCl, pH 7.8) equilibrated Ni-NTA resin to capture uncleaved protein, His_6_-TEV protease and cleaved His_6_-tag. The flow-through was then concentrated to a final volume of 1 mL using 3.5 kDa MWCO spin filter (GE Healthcare) and applied to a HiLoad 16/600 Superdex 75 pg column (GE Healthcare). The column was equilibrated with 1.5 column-volumes of column buffer (20 mM HEPES, pH 7.5, 500 mM NaCl) at 1 mL/min. The prep was analyzed by SDS-PAGE and desirable fractions concentrated to 10–15 mg/mL, prior to storage at −80 °C.

### Expression of Pfs25-SpyTag

Suspension HEK293E cells were cultured to 2 million cells/mL in Expi293 Expression Medium (Life Technologies) containing penicillin/streptomycin at 37 °C and 8% CO_2_ at 125 rpm. Cells were transiently transfected with 1 μg plasmid complexed to polyethylenimine per million cells. Supernatant was harvested after 72 h. Pfs25-SpyTag was purified by CaptureSelect C-tag Affinity Matrix (Life Technologies) using the manufacturer’s instructions, followed by size-exclusion chromatography (HiLoad 16/600 Superdex 200 pg column, GE Healthcare).

### SpyCatcher-VLP:SpyTag-protein interaction

SpyTag-MBP and SpyTag DA-MBP were expressed in *E. coli* and purified by Ni-NTA as previously described^33^. SpyCatcher-VLPs were reacted with 1.5× molar excess of SpyTag interaction partner in a total volume of 150 μL including 15 μL of 10× reaction buffer (40 mM Na_2_HPO_4_, 200 mM sodium citrate, pH 6.2); if the volume of SpyCatcher-VLPs and SpyTag-partner did not amount to 150 μL or no partner was added for the control, the difference in volume was topped up with 20 mM Tris•HCl, 300 mM NaCl, pH 7.8. Protein concentrations for partners were: SpyTag-MBP, 82.5 μM; SpyTag DA-MBP, 87 μM; SpyTag-CIDR(HB3var03), 144.3 μM; SpyTag-CIDR(IT4var07, TEV cleaved), 263.5 μM; Pfs25-SpyTag, 59μM. The reaction was performed for 3 h at RT and stopped with 6 × SDS-PAGE loading buffer [20% glycerol, 100 mM Tris•HCl, 4% SDS, 0.2% bromophenol blue, 1% (v/v) 2-mercaptoethanol, pH 6.8] and subsequent heating for 3 min at 95 °C.

### Peptides

Peptides were synthesized by Insight Biotechnology at >95% purity, validated by HPLC and mass spectrometry. SpyTag-Telo is SpyTag linked to a human telomerase reverse transcriptase mutant epitope implicated with increased risk of various cancers^43^ (biotin-GAHIVMVDAYKPTREARPALLTSRLRFIPK). SpyTag-Telo was dissolved at 100 mg/mL in a 2:1 mixture of H_2_O to acetic acid. Stock solution was diluted 10-fold with 50 mM glycine, 25 mM sodium citrate, 0.1% (v/v) Tween 20, pH 8.0 buffer, prior to conjugation. EGFRvIII-SpyTag contains the fusion junction epitope of human epidermal growth factor receptor (EGFR) found in glioblastoma^44^ (LEEKKGNYVVTDHGAHIVMVDAYKPTK–biotin). This peptide was dissolved at 100 mg/mL in a 1:4 mixture of formic acid to H_2_O. EGFRvIII-SpyTag stock was diluted 10-fold with 10 × reaction buffer (40 mM Na_2_HPO_4_, 200 mM sodium citrate, pH 6.2), prior to conjugation.

### SpyCatcher-VLP:SpyTag-peptide reaction

SpyCatcher-VLPs were expressed and purified as before, except washing was with NTA buffer (50 mM Tris•HCl, 300 mM NaCl, pH 7.8, 0.1% v/v Tween 20) plus 200 mM imidazole and elution was with NTA buffer plus 2 M imidazole. No dialysis was performed prior to reaction. SpyCatcher-VLPs at 2.9 mg/mL were mixed with a 2.4 × molar excess of SpyTag-Telo or a 2.7 × molar excess of EGFRvIII-SpyTag (as compared to the concentration of SpyCatcher moieties). After addition of 10 × reaction buffer (40 mM Na_2_HPO_4_, 200 mM sodium citrate, pH 6.2), the sample was incubated for 3 h at 22 °C. To control for any change in the buffer altering the gel mobility of SpyCatcher-VLPs, the “Solvent control” sample contained SpyCatcher-VLPs mixed for 3 h at RT with a solution matching the solvent concentration of the peptide sample (acetic acid for SpyTag-Telo or formic acid for EGFRvIII-SpyTag), with no reaction buffer added. The “Solvent + Rxn control” sample was the same as the “Solvent control” sample, except that phosphate-citrate reaction buffer was included. The reaction was stopped by adding 6× SDS-PAGE loading buffer and heating for 3 min at 95 °C. Samples were analyzed by 14% SDS-PAGE with Coomassie staining.

### SDS-PAGE

SDS-PAGE was performed on 12% Tris-glycine gels using an XCell SureLock system (Life Technologies) unless otherwise indicated. Gels were stained with InstantBlue (Expedeon) and imaged using a ChemiDoc XRS imager and QuantityOne (version 4.6) software (Bio-Rad).

Protein concentrations were determined using a bicinchoninic acid assay (Pierce™ BCA Protein Assay Kit, Thermo Scientific), following manufacturer’s instructions without the use of reducing agent.

### Size-exclusion chromatography (SEC)

VLPs (5 mL at A280 ~7) eluted from Ni-NTA resin were applied to a previously equilibrated HiPrep 16/60 Sephacryl S-500 HR (GE Healthcare) on a fast protein liquid chromatography (FPLC) system Purifier 10 (GE Healthcare). The mobile-phase column buffer was 20 mM Tris•HCl, 1 M NaCl, pH 8.5 and the applied flow-rate was 1.0 mL/min, all at 4 °C. For calibration, a high molecular weight gel filtration standard (Bio-Rad) was used.

### Agarose gel electrophoresis

A 1% (w/v) agarose gel, prepared in 40 mM Tris, 20 mM acetic acid, 1 mM EDTA, pH 8.0, containing 1 × SybrSafe (Life Technologies) according to manufacturer’s instruction was loaded with 20 μL SpyCatcher-VLPs, with or without 30 μM SpyTag-CIDR(IT4var07) conjugation, after mixing with DNA loading buffer. DNA loading buffer was 0.15 g/mL Ficoll 400 (Sigma), 40 mM Tris, 20 mM acetic acid, 1 mM EDTA, pH 8.0 supplemented with Orange G (Sigma). The samples were run for 1 h at 120 V (7.7 V/cm) in TAE (40 mM Tris, 20 mM acetic acid, 1 mM EDTA, pH 8.0). First SybrSafe was imaged on a UV table equipped with an XcitaBlue™ screen kit for excitation with blue light. Then the gel was stained with a Coomassie-based dye, InstantBlue, and imaged with visible light illumination using a ChemiDoc XRS imager and QuantityOne (version 4.6) software.

### Transmission electron microscopy (TEM)

10 μL SpyCatcher-VLPs (0.2 mg/mL) were applied to freshly glow-discharged carbon 200 mesh copper grids for 2 min, blotted with filter paper, and stained with 2% uranyl acetate for 10 s, then blotted and air dried. Grids were imaged in a FEI Tecnai T12 transmission electron microscope at 120 kV using a Gatan US1000 CCD camera. Particle diameter for each group was measured with FIJI (ImageJ) with 2 nm bin size (n = 100).

### Preparation of immunogens

For CIDR immunization, SpyCatcher-VLPs (30 μM) were incubated with 1.5 × molar excess of SpyTag-CIDR (IT4var07, TEV cleaved to remove His_6_-tag) for 3 h at RT with addition of 10 × reaction buffer (40 mM Na_2_HPO_4_, 200 mM sodium citrate, pH 6.2). The reaction volume was then dialyzed with 300 kDa cut-off membrane twice against 1,000-fold excess PBS with 0.1% (v/v) Tween 20 to remove unreacted SpyTag-CIDR. Doses were matched to 20 μg CIDR(IT4var07)-equivalent for the SpyCatcher-VLPs + untagged CIDR(IT4var07) group and the CIDR(IT4var07)-alone group and diluted with sterile endotoxin-free PBS (Life Technologies) with 0.1% (v/v) Tween 20 to a total of 50 μL per dose. Two individual batches prepared within 24 h prior to immunization were 0.22 μm-filtered, using autoclaved polyvinylidene difluoride (PVDF) membrane (Whatman).

Endotoxin concentrations were determined with Pierce LAL Chromogenic Endotoxin Quantitation Kit (Thermo Fisher Scientific) following manufacturer’s instructions and were below 1 Endotoxin Unit/mL for SpyCatcher-VLP preparations.

For Pfs25 immunization, SpyCatcher-VLPs (30 μM) were incubated with 1.5 × molar excess of Pfs25-SpyTag for 3 h at RT with addition of 10 × reaction buffer (40 mM Na_2_HPO_4_, 200 mM sodium citrate, pH 6.2). The reaction was then dialyzed with 300 kDa cut-off membrane three times against 1,000-fold excess 50 mM glycine, 25 mM sodium citrate, 0.1% (v/v) Tween 20 to remove unreacted Pfs25-SpyTag. Doses were matched to 2.5 μg Pfs25 equivalent for each group and diluted with sterile 50 mM glycine, 25 mM sodium citrate, 0.1% (v/v) Tween 20 to a total of 50 μL per dose or 25 μL per dose in groups containing adjuvant. Adjuvanted groups were mixed 1:1 with stock AddaVax (InvivoGen, distributed by Source BioScience) with gentle pipetting and kept on ice until immunization within 2 h. An AddaVax adjuvanted negative control group for microscopy was prepared in the same manner, replacing Pfs25 with ovalbumin as the immunogen.

### Immunizations

Animal experiments and procedures were performed according to the UK Animals (Scientific Procedures) Act Project License (PPL 30/2414 and 30/2889) and approved by the Oxford University Local Ethical Review Body. Age-matched (~60 days old at first immunization) female BALB/c mice (Harlan, UK), housed in specific-pathogen free environments, were injected intramuscularly with protein sample into each rear leg using a protein prime-boost regime. Protein samples for injection were prepared in sterile endotoxin-free PBS (Thermo Fisher Scientific) with 0.1% (v/v) Tween 20. Mice received the priming dose on day 0, followed by a boosting dose on the indicated day. Blood samples were taken to obtain sera for endpoint ELISA. Sera were obtained from whole blood by leaving samples overnight at 4 °C to clot, followed by 5 min centrifugation at 16,000 *g* in a benchtop centrifuge at RT. Sera were pipetted into fresh Eppendorf tubes with care not to disturb the pellet. 6 mice were used per condition.

### Endpoint ELISA

Nunc-Immuno MaxiSorp plates (Thermo Scientific) were coated with 100 μL/well of CIDR(IT4var07) or SpyCatcher-VLP at 1 μg/mL in coating buffer (15 mM sodium carbonate with 35 mM sodium bicarbonate) overnight at 4 °C. Plates were washed with PBS/T (PBS supplemented with 0.5% Tween 20) and blocked for 1 h at RT with 10% skimmed milk in PBS/T. Serum samples were added in duplicates and diluted 3-fold down the plate, followed by incubation for 2 h at RT and then washed with PBS/T as before. Goat anti-mouse total IgG conjugated to alkaline phosphatase (Sigma-Aldrich) was added to the plate (1:3,000 dilution in PBS/T) for 1 h at RT. After a final wash in PBS/T, p-nitrophenylphosphate (Sigma-Aldrich) diluted in diethanolamine buffer (1 M diethanolamine, pH 9.8) (Thermo Scientific) was used as a developing substrate. A405 was obtained using an ELx800 absorbance microplate reader (Biotek). The endpoint titer is defined as the x-axis intercept of the dilution curve at an absorbance value (±3 s.d.) greater than the A405 for a serum sample from a mouse immunized with an irrelevant protein control, Pfs25-nanoparticle from a previous experiment.

ELISA against Pfs25 was performed according to a standardized protocol using a reference serum, as described previously^70^. Nunc-Immuno MaxiSorp plates were coated with Pfs25 protein at 0.1 μg per well in PBS/T overnight at RT. Plates were washed with PBS/T and blocked for 1 h with 5% skimmed milk in PBS/T at RT. Test serum samples were diluted in PBS/T and 100 μL of each were added to the relevant well, followed by incubation for 2 h at RT, and then washing with PBS/T six times at RT. Donkey anti-mouse total IgG conjugated to alkaline phosphatase (Jackson ImmunoResearch Laboratories) was added to the plate (1:3,000 dilution in PBS) for 1 h at RT. After a final wash in PBS/T, samples were developed with p-nitrophenylphosphate and measured as described above. All samples were tested against a serially-diluted standard reference serum with a known antibody titer, prepared as described previously^71^. The absorbance of individual test samples was converted into Pfs25 Antibody Units using a standard curve generated by this standard reference serum as described^70^.

### Statistical analysis of immunizations

Statistical analyses were performed using GraphPad Prism. Continuous variables between two groups were compared by a two-tailed Mann-Whitney test.

### Immunofluorescence

Immunofluorescence was performed on ookinetes fixed with paraformaldehyde (PFA) as described previously^63^. Ookinete culture smears of Pfs25DR3 transgenic *Plasmodium berghei* parasite (a chimeric *P. berghei* line which expresses Pfs25 in place of the endogenous Pbs25, provided by Dr Andrew Blagborough, Imperial College London) were fixed for 10 min in PBS with 4% PFA at RT, followed by washing in PBS. Slides were blocked for 1 h in PBS with 3% (w/v) BSA at RT, followed by incubation for 45 min with 2 μg/mL anti-Pfs25 monoclonal antibody 4B7^72^ (a kind gift from Kazutoyo Miura, National Institutes of Health, Maryland, USA) or test antiserum (1:100, from mice primed and boosted with AddaVax and SpyCatcher-VLPs:Pfs25-SpyTag or Ovalbumin) in PBS with 3% (w/v) BSA at RT in a humidified chamber. Slides were washed three times in PBS and then incubated with AlexaFluor 488-conjugated goat anti-mouse IgG (1:500) (Thermo Fisher Scientific) in PBS with 3% (w/v) BSA at RT for 45 min. After another three washes with PBS, slides were mounted with mounting medium with DAPI (Vector Laboratories), allowed to set overnight at 4 °C and analyzed by wide-field fluorescence microscopy on a DMI3000 B microscope (Leica Microsystems). All samples were stained, imaged and analyzed under the same conditions.

## Additional Information

**How to cite this article**: Brune, K. D. *et al.* Plug-and-Display: decoration of Virus-Like Particles via isopeptide bonds for modular immunization. *Sci. Rep.* **6**, 19234; doi: 10.1038/srep19234 (2016).

## Supplementary Material

Supplementary Information
