# Supplementary material for: Plug-and-Display: decoration of Virus-Like Particles via isopeptide bonds for modular immunization
Source: Sci Rep. 2016 Jan 19;6:19234. doi: 10.1038/srep19234 (PMC4725971; doi:10.1038/srep19234)
Supplement: Supplementary Information [file srep19234-s1.pdf]

Supplementary Information
Plug-and-Display: decoration of Virus-Like Particles via isopeptide bonds for modular immunization

Karl D. Brune[1], Darren B. Leneghan[2], Iona J. Brian[2], Andrew S. Ishizuka[2], Martin F. Bachmann[2,3], Simon J. Draper[2], Sumi Biswas[2] & Mark Howarth[1]

[1]Department of Biochemistry, University of Oxford, South Parks Road, Oxford, OX1 3QU, UK. [2]Jenner Institute, University of Oxford, Oxford, OX3 7DQ, UK. [3]University Institute of Immunology, University of Bern, Sahli Haus 2, Inselspital, Bern, CH-3010, Switzerland.

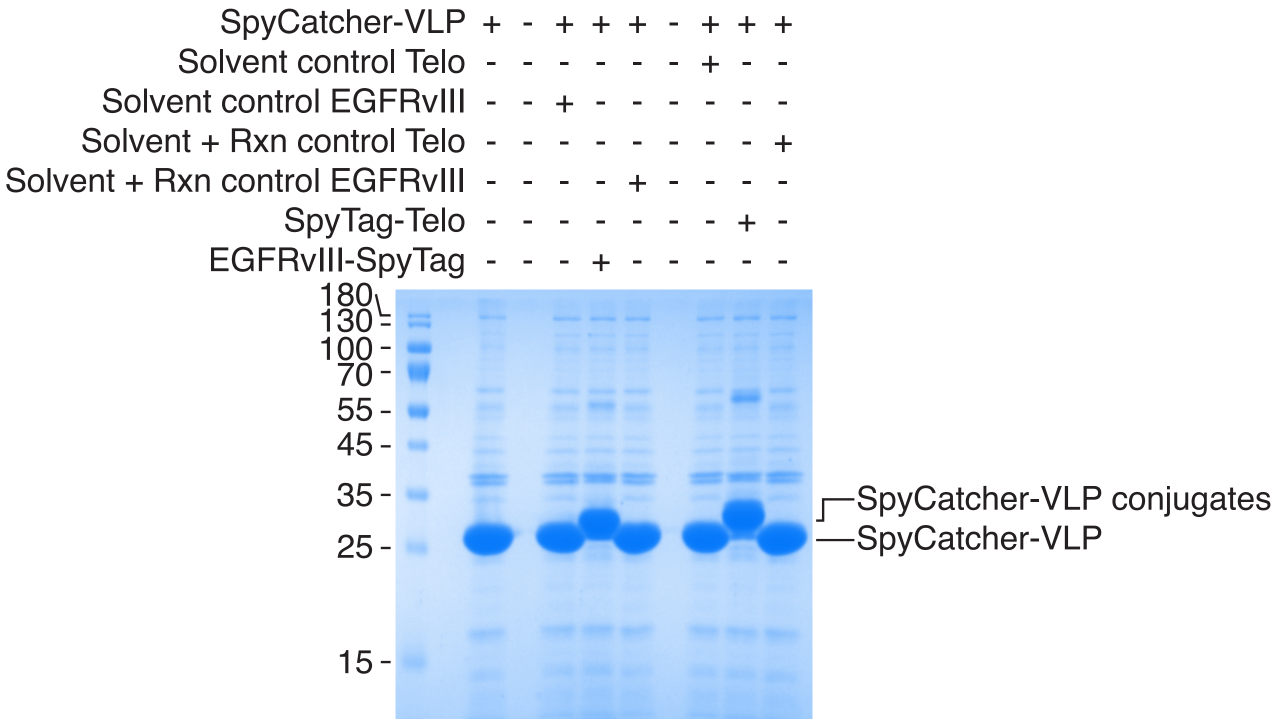

Supplementary Figure S1. SpyCatcher-VLPs reacted efficiently with SpyTag-linked peptides. SpyCatcher-VLPs were incubated for 3 h at 22 °C with the synthetic peptides SpyTag-Telo or EGFRvIII-SpyTag. Control incubations of SpyCatcher-VLPs with solvent or solvent plus reaction buffer (Solvent + Rxn) appropriate to each peptide were also performed. Samples were then boiled in SDS-loading buffer and analyzed by SDS-PAGE with Coomassie staining.

a

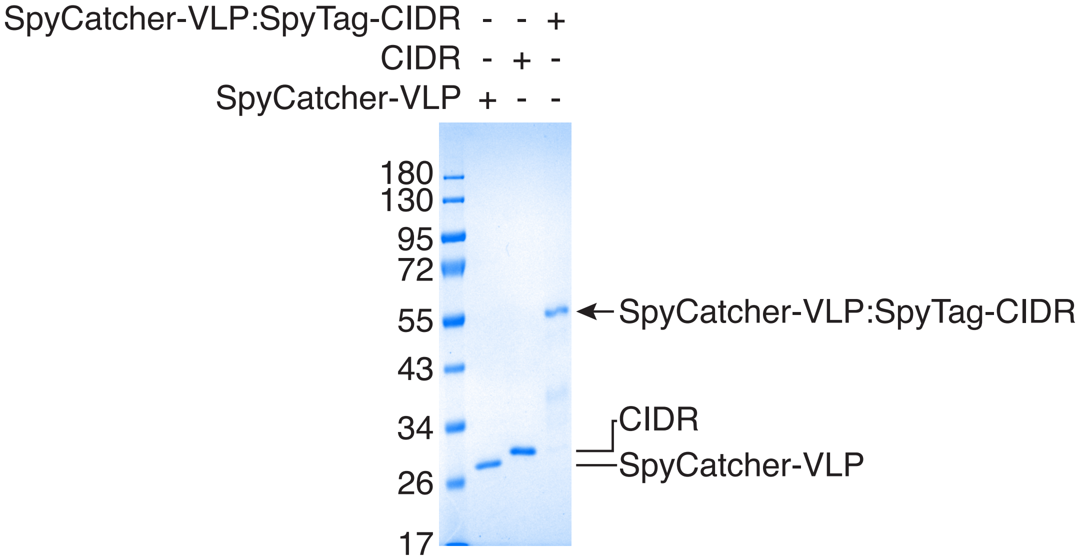

b

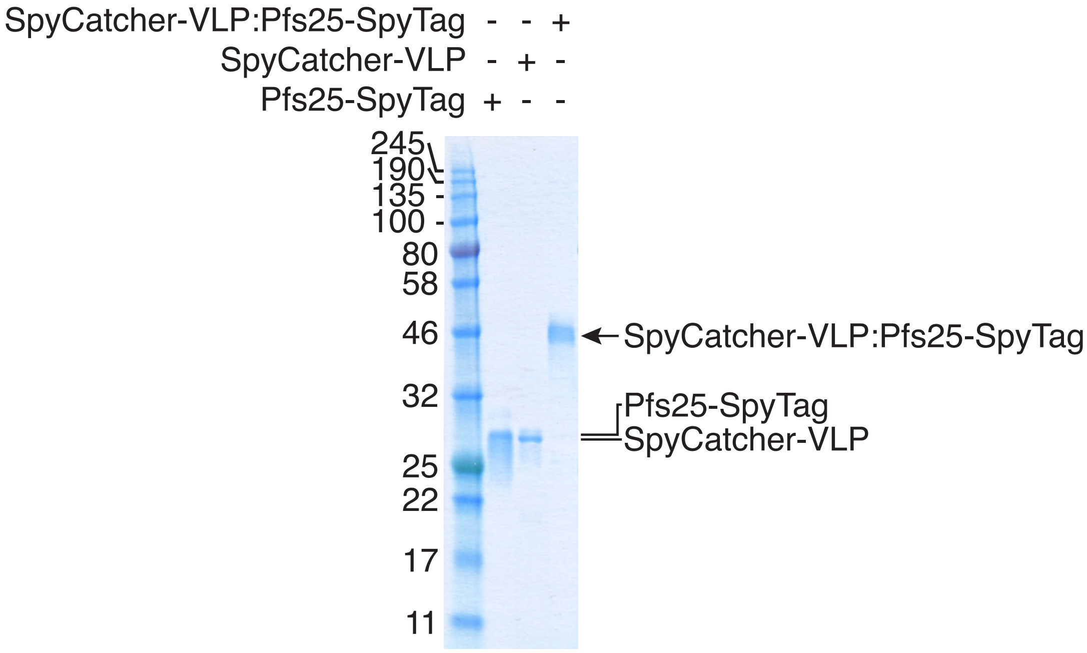

Supplementary Figure S2. SDS-PAGE on conjugates used for immunization. (a) SpyCatcher-VLP:SpyTag-CIDR conjugation. SpyCatcher-VLPs, CIDR(IT4var07) and the purified SpyCatcher-VLP:SpyTag-CIDR(IT4var07) conjugate were boiled in SDS-loading buffer and analyzed by SDS-PAGE with Coomassie staining. (b) SpyCatcher-VLP:Pfs25-SpyTag conjugation. SpyCatcher-VLPs, Pfs25-SpyTag and the purified SpyCatcher-VLP:Pfs25-SpyTag conjugate were boiled in SDS-loading buffer and analyzed by SDS-PAGE with Coomassie staining.

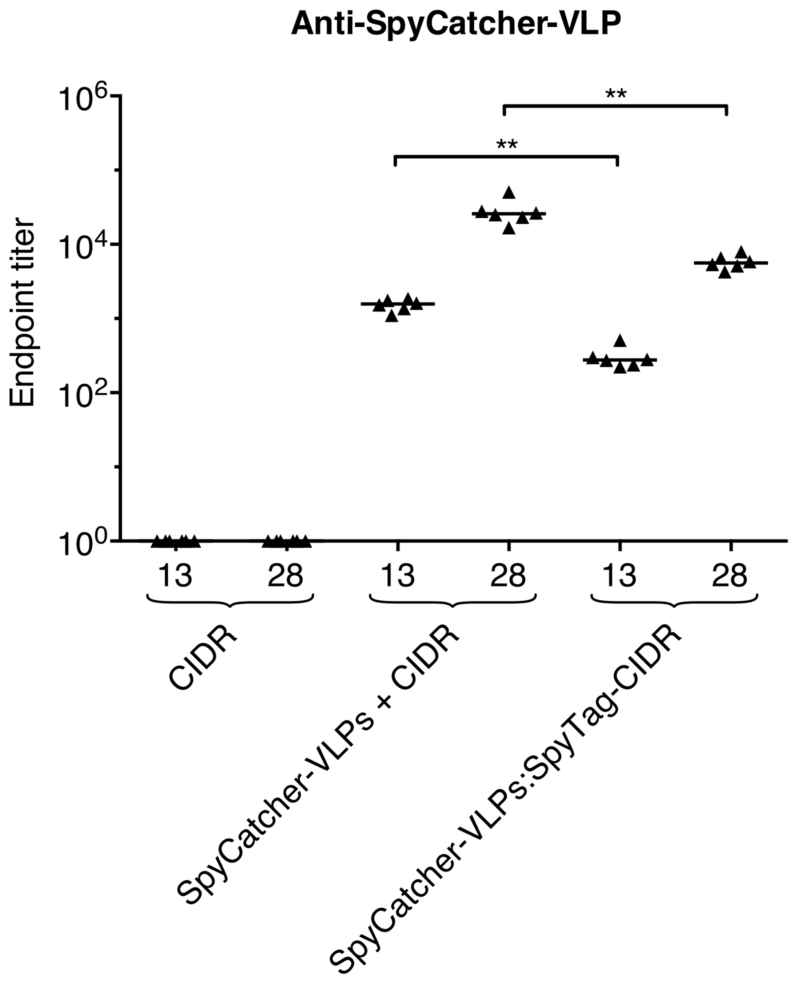

Supplementary Figure S3. Antibody response to SpyCatcher-VLP after immunization. 6 mice per condition were immunized on days 0 and 14 with CIDR, SpyCatcher-VLPs mixed with CIDR, or SpyCatcher-VLPs covalently conjugated to SpyTag-CIDR. The total IgG anti-SpyCatcher-VLP titer was determined by ELISA at day 13 or 28, by coating the ELISA plate with purified SpyCatcher-VLP. Triangles represent the value for each mouse, while the horizontal bars represent the medians for each group. ** $p<0.01$, determined by Mann-Whitney test (n=6).
